# Contributions of Limb Joints to Energy Absorption during Landing in Cats

**DOI:** 10.1155/2019/3815612

**Published:** 2019-08-18

**Authors:** Xueqing Wu, Baoqing Pei, Yuyang Pei, Nan Wu, Kaiyuan Zhou, Yan Hao, Wei Wang

**Affiliations:** ^1^School of Biological Science and Medical Engineering, Beihang University, Beijing 100083, China; ^2^Beijing Advanced Innovation Centre for Biomedical Engineering, Beihang University, Beijing 100083, China; ^3^School of Public Health, Nanjing Medical University, Nanjing 211166, China

## Abstract

There is a high risk of serious injury to the lower limbs in a human drop landing. However, cats are able to jump from the same heights without any sign of injury, which is attributed to the excellent performance of their limbs in attenuating the impact forces. The bionic study of the falling cat landing may therefore contribute to improve the landing-shock absorbing ability of lower limbs in humans. However, the contributions of cat limb joints to energy absorption remain unknown. Accordingly, a motion capture system and plantar pressure measurement platform were used to measure the joint angles and vertical ground reaction forces of jumping cats, respectively. Based on the inverse dynamics, the joint angular velocities, moments, powers, and work from different landing heights were calculated to expound the synergistic mechanism and the dominant muscle groups of cat limb joints. The results show that the buffering durations of the forelimbs exhibit no significant difference with increasing height while the hindlimbs play a greater role than the forelimbs in absorbing energy when jumping from a higher platform. Furthermore, the joint angles and angular velocities exhibit similar variations, indicating that a generalized motor program can be adopted to activate limb joints for different landing heights. Additionally, the elbow and hip are recognized as major contributors to energy absorption during landing. This experimental study can accordingly provide biological inspiration for new approaches to prevent human lower limb injuries.

## 1. Introduction

Cats are generally acknowledged to have excellent landing buffering capacities, achieved through natural selection, and have accordingly received significant scientific attention. A number of cases have been studied, finding that the death rate of cats is less than 10% after falling from a high rise [1]. As the saying goes: cats have nine lives, emphasizing the fact that cats indeed have an extraordinary ability to survive falls. In terms of this phenomenon, a number of researchers have studied the body posture of cats as they fall, using high-speed cameras, and the results show that falling cats make gyroscopic turns such that their forelimbs and hindlimbs land successively, regardless of the cat's orientation at the start of the fall [2–4]. Therefore, it has been suggested that cat limbs play a significant role in dissipating the impact forces during landing.

From a biomechanical point of view, external and internal forces can be mediated by manipulating the limb joint kinematics and limb muscle groups that contribute to the reduction and transfer of mechanical energy. By using strain gauge-based force transducers, it has been shown that the force and activity patterns of the gastrocnemius (GA), soleus (SO), and plantaris (PL) muscles are beneficial for transferring mechanical energy between adjacent joints during locomotion [5–7]. Additionally, the activation of cat hindlimb muscles is directed opposite to the endpoint reaction forces of these muscles [8]. Similarly, some studies have suggested that the activity of limb muscles is critical in the cat landing process and that this activity is determined by the jumping condition. For example, to avoid injury, limb muscles become tensed before touching the ground, and the magnitude and timing of prelanding limb muscle activity are adjusted to be appropriate for the jump height [9]. Meanwhile, the responses of the elbow extensors to ground reaction forces (GRFs) have been studied, showing that, for a given cat, both the vertical and horizontal GRFs increase with jump height while torque values at the elbow joint do not change significantly [10]. Furthermore, a study on reflexes in cat ankle muscles indicates that large and rapid reflexes indeed occur during landing and the lengths of the ankle extensors begin to increase only after the toes have developed significant dorsal flexion [11]. Additionally, the manner of distribution of impact forces between the forelimbs and hindlimbs of cats has been found to be related to the jump height, and the hindlimbs have been found to play an increasing role in the absorption of energy with increasing jump height [12].

As stated above, there is currently sufficient evidences to elucidate the role of cat limbs in energy absorption based on limb kinematics, kinetics, and EMG responses of muscles. However, the contributions of various limb joint muscle groups to the total energy absorption during landing remain unknown. It is known that falling from different heights results in the adoption of different cat limb control strategies in order to effectively attenuate the impact forces. Studies of joint energy absorption strategies under different jump heights can thus provide comprehensive insight into the internal buffering mechanism of cats during landing. In the previous studies, the GRFs were measured using one or two AMTI force plates, but this method could not be used to differentiate between the GRFs of the forelimbs and hindlimbs of the cats. As a result, it is not well established that this information is sufficient to provide representative calculations of the mechanical energy absorbed by the joint muscles of cat limbs.

The objective of this paper is therefore to study the contributions of different limb joints to energy absorption and to further understand the energy dissipation strategies of joint muscles during landing in cats. In this study, we conducted experiments in which domestic cats self-initiated jumps from different heights and the vertical ground reaction forces (VGRFs) as well as the kinematic (joint angles and angular velocities), kinetic (joint moments and joint powers), and energetic (joint work) data were analyzed based on the planar dynamics and inverse dynamics. Additionally, the synergistic mechanism of cat joints was described, making it possible to visualize the events that occur during the cat landing process. The results of this study will help to interpret and understand the role of dominant joints in energy absorption. They will also promote the understanding of the internal buffering mechanisms of cat limbs during landing. A more practical motivation for this study is to provide useful information for the future development of high-efficiency buffering and energy absorption equipment.

## 2. Materials and Methods

### 2.1. Animal Training and Experimental Protocol

Five healthy adult domestic cats (2.45 ± 0.29 years of age, 3.6 ± 0.35 kg) were trained to jump down onto a MatScan (Texscan Inc.) from an adjustable platform of height between 1 m and 2 m. Training was conducted for about half an hour, five times a week over three weeks before the landing experiments were conducted. After each successful training experiment, the cat was given food as a reward. During the six experimental sessions, at least five jumps per height were recorded in random increments of 0.2 m between 1 m and 2 m, and the cat was given enough rest after each jump to ensure that the results were not affected by physical condition, adaptability, etc. All experimental procedures were approved by the Science and Ethics Committee of Beihang University.

### 2.2. Data Measurement and Analysis

In this study, we investigated only the distribution of the *vertical* ground reaction forces (VGRFs) between the limb joints, as the forces in the *mediolateral* and *fore-aft* directions are small enough to be ignored [12]. In order to accurately compare the energy absorption of the forelimbs and hindlimbs, a single MatScan (150 Hz; Texscan Inc., South Boston, MA, USA) was used to select and measure only the VGRFs of the right fore (RF) and right hind (RH) limbs from the impact on the mat. All raw VGRF data for an individual were scaled to multiples of body weight (BW) for each cat. In particular, the VGRFs of the RF limbs, displayed as two-dimensional images, were used to determine the buffering durations of the forelimbs, defined as beginning with the touchdown of the RF paws and ending at the time at which the RF wrists began to leave the MatScan.

**Figure 1:**
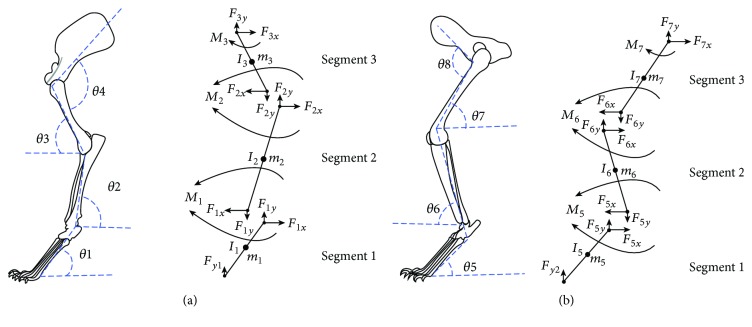
The left side shows the angles of the forelimb (a) and hindlimb (b) used in the equations, and the right side shows free-body diagrams of the same limbs, in which the reaction forces and moments acting on each joint are indicated.

Before the experiment, the areas of interest on the RF and RH limbs were shaved. Reflective markers with a diameter of 9 mm were then placed over the shoulder blade, shoulder, elbow, wrist joint, and fingertip of the RF limb and the pelvis, hip, knee, ankle joint, and toe of the RH limb to obtain the eight angles shown in Figure 1. A motion capture system (100 Hz; Vicon Inc., Denver, CO, USA), synchronized to the MatScan, was used to collect the positions of these markers. The buffering durations of the hindlimbs were defined as beginning with the touchdown of the hind paws and ending with peak knee flexion. During the experiment, it was found that the slippage of markers on the elbow and knee joints was quite serious. In order to diminish measurement artifacts caused by this slippage, an optimization procedure written in MATLAB was used to calculate the positions of the elbow and knee joints, which were then optimized using constraints to be closest to the collected positions of elbow and knee joint markers. The constraint placed on the elbow joint in the optimization procedure, for example, was that the distance from the calculated elbow joint to the collected shoulder and wrist joint be the same as the premeasured arm and forearm length, respectively.

The limb of each cat was assumed to be a planar link-segment rigid body model. Segment parameters, including segment mass and moment of inertia, obtained from a previous study [13] and combined with the kinematic data and VGRFs, were imported into MATLAB to calculate the internal joint moment for each joint based on the inverse solution. Joint muscle power was defined as the product of the internal joint moment and joint angular velocity, calculated as the rate of change of angular displacement. The displacement and angular velocity data were smoothed using a five-piece moving arc to further reduce measurement artifacts [10]. The integral of joint muscle power over the buffering time determined the joint work used to represent the energy absorbed by a given joint. All joint moments, muscle powers, and work were expressed in units of Nm/Kg, W/Kg, and J/Kg, respectively.

### 2.3. Inverse Dynamics Analysis

**Figure 2:**
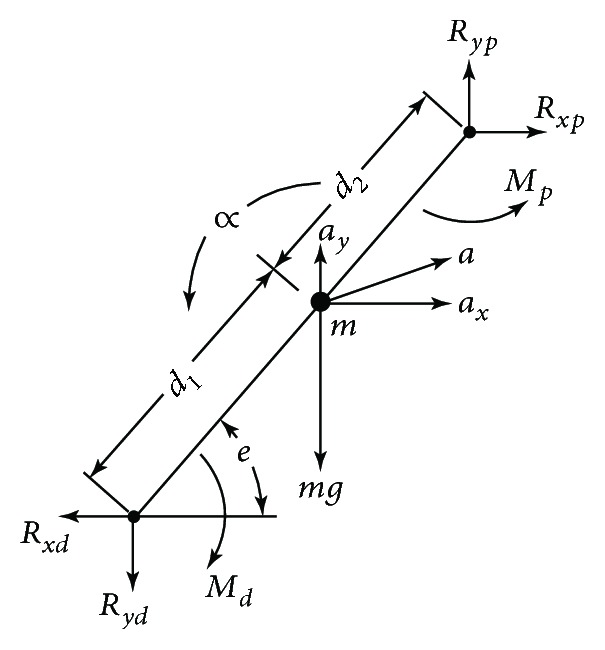
Complete free-body diagram of a single limb segment, showing the reaction and gravitational forces, net moments of force, and all linear and angular accelerations.

Each limb segment was assumed to act independently under a combination of joint reaction forces, joint muscle moments, and gravity, as illustrated in Figure 2.

Based on Figure 2 [14], the following equations can be obtained:

(1) $$\begin{array}{c} \begin{array}{c} \sum F_{x}={ma}_{x}=R_{xp}-R_{xd},\\ \sum F_{y}={ma}_{y}=R_{yp}-R_{yd}-mg,\\ \sum M=I_{0}\alpha , \end{array} \end{array}$$

where *F*_*x*_ and *F*_*y*_ are the forces in the X and Y directions, respectively; *m* is the segment mass; *a*_*x*_ and *a*_*y*_ are the X and Y components of acceleration of the segment center of mass (COM), respectively; *M* is the moment about the segment; and *l*_0_ and *α* are the moment of inertia and angular acceleration of the segment in the plane of movement, respectively.

The RF and RH limbs of the subject cats were analyzed by splitting each into three rigid links. At the same time, the COM was assumed to be at the midpoint of a segment. Therefore, based on equation (1), the joint moments in the three segments of the RF and RH limbs for each cat in this paper can be calculated as follows.

For the RF limb

Segment 1

(2) $$\begin{array}{c} \left\{\begin{array}{c} F_{1x}=m_{1}\left(-\frac{L_{1}}{2}{{\dot{\theta }}_{1}}^{2}\cos\theta_{1}-\frac{L_{1}}{2}{\ddot{\theta }}_{1}\sin\theta_{1}\right),\\ F_{y1}+F_{1y}-m_{1}g=m_{1}\left(-\frac{L_{1}}{2}{{\dot{\theta }}_{1}}^{2}\sin\theta_{1}+\frac{L_{1}}{2}{\ddot{\theta }}_{1}\cos\theta_{1}\right),\\ M_{1}+F_{1x}\frac{L_{1}}{2}\sin\theta_{1}+F_{y}\frac{L_{1}}{2}\cos\theta_{1}-F_{1y}\frac{L_{1}}{2}\cos\theta_{1}=-I_{1}{\ddot{\theta }}_{1} \end{array}\right. \end{array}$$

Segment 2

(3) $$\begin{array}{c} \left\{\begin{array}{c} F_{2x}-F_{1x}=m_{2}\left[-L_{1}\left({{\dot{\theta }}_{1}}^{2}\cos\theta_{1}+{\ddot{\theta }}_{1}\sin\theta_{1}\right)-\frac{L_{2}}{2}\left({{\dot{\theta }}_{2}}^{2}\cos\theta_{2}+{\ddot{\theta }}_{2}\sin\theta_{2}\right)\right],\\ F_{2y}-F_{1y}-m_{2}g=m_{2}\left[L_{1}\left(-{{\dot{\theta }}_{1}}^{2}\sin\theta_{1}+{\ddot{\theta }}_{1}\cos\theta_{1}\right)+\frac{L_{2}}{2}\left(-{{\dot{\theta }}_{2}}^{2}\sin\theta_{2}+{\ddot{\theta }}_{2}\cos\theta_{2}\right)\right],\\ M_{2}-M_{1}+F_{2x}\frac{L_{2}}{2}\sin\theta_{2}-F_{2y}\frac{L_{2}}{2}\cos\theta_{2}+F_{1x}\frac{L_{2}}{2}\sin\theta_{2}-F_{1y}\frac{L_{2}}{2}\cos\theta_{2}=-I_{2}{\ddot{\theta }}_{2} \end{array}\right. \end{array}$$

Segment 3

(4) $$\begin{array}{c} \left\{\begin{array}{c} F_{3x}-F_{2x}=m_{3}\left[-L_{1}\left({{\dot{\theta }}_{1}}^{2}\cos\theta_{1}+{\ddot{\theta }}_{1}\sin\theta_{1}\right)-L_{2}\left({{\dot{\theta }}_{2}}^{2}\cos\theta_{2}+{\ddot{\theta }}_{2}\sin\theta_{2}\right)+\frac{L_{3}}{2}\left({{\dot{\theta }}_{3}}^{2}\cos\theta_{3}+{\ddot{\theta }}_{3}\sin\theta_{3}\right)\right],\\ F_{3y}-F_{2y}-m_{3}g=m_{3}\left[L_{1}\left(-{{\dot{\theta }}_{1}}^{2}\sin\theta_{1}+{\ddot{\theta }}_{1}\cos\theta_{1}\right)+L_{2}\left(-{{\dot{\theta }}_{2}}^{2}\sin\theta_{2}+{\ddot{\theta }}_{2}\cos\theta_{2}\right)+\frac{L_{3}}{2}\left(-{{\dot{\theta }}_{3}}^{2}\sin\theta_{3}+{\ddot{\theta }}_{3}\cos\theta_{3}\right)\right],\\ M_{3}-M_{2}+F_{3x}\frac{L_{3}}{2}\sin\theta_{3}+F_{3y}\frac{L_{3}}{2}\cos\theta_{3}+F_{2x}\frac{L_{3}}{2}\sin\theta_{3}+F_{2y}\frac{L_{3}}{2}\cos\theta_{3}=I_{3}{\ddot{\theta }}_{3} \end{array}\right. \end{array}$$

For the RH limb

Segment 1

(5) $$\begin{array}{c} \left\{\begin{array}{c} F_{5x}=m_{5}\left(-\frac{L_{5}}{2}{{\dot{\theta }}_{5}}^{2}\cos\theta_{5}-\frac{L_{5}}{2}{\ddot{\theta }}_{5}\sin\theta_{5}\right),\\ F_{y2}+F_{5y}-m_{5}g=m_{5}\left(-\frac{L_{5}}{2}{{\dot{\theta }}_{5}}^{2}\sin\theta_{5}+\frac{L_{5}}{2}{\ddot{\theta }}_{5}\cos\theta_{5}\right),\\ M_{5}+F_{5x}\frac{L_{5}}{2}\sin\theta_{5}+F_{y}\frac{L_{5}}{2}\cos\theta_{5}-F_{5y}\frac{L_{5}}{2}\cos\theta_{5}=-I_{5}{\ddot{\theta }}_{5} \end{array}\right. \end{array}$$

Segment 2

(6) $$\begin{array}{c} \left\{\begin{array}{c} F_{6x}-F_{5x}=m_{6}\left[-L_{5}\left({{\dot{\theta }}_{5}}^{2}\cos\theta_{5}+{\ddot{\theta }}_{5}\sin\theta_{5}\right)+\frac{L_{6}}{2}\left({{\dot{\theta }}_{6}}^{2}\cos\theta_{6}+{\ddot{\theta }}_{6}\sin\theta_{6}\right)\right],\\ F_{6y}-F_{5y}-m_{6}g=m_{6}\left[L_{5}\left(-{{\dot{\theta }}_{5}}^{2}\sin\theta_{5}+{\ddot{\theta }}_{5}\cos\theta_{5}\right)+\frac{L_{6}}{2}\left(-{{\dot{\theta }}_{6}}^{2}\sin\theta_{6}+{\ddot{\theta }}_{6}\cos\theta_{6}\right)\right],\\ M_{6}-M_{5}+F_{6x}\frac{L_{6}}{2}\sin\theta_{6}+F_{6y}\frac{L_{6}}{2}\cos\theta_{6}+F_{5x}\frac{L_{6}}{2}\sin\theta_{6}+F_{5y}\frac{L_{6}}{2}\cos\theta_{6}=I_{6}{\ddot{\theta }}_{6} \end{array}\right. \end{array}$$

Segment 3

(7) $$\begin{array}{c} \left\{\begin{array}{c} F_{7x}-F_{6x}=m_{7}\left[-L_{5}\left({{\dot{\theta }}_{5}}^{2}\cos\theta_{5}+{\ddot{\theta }}_{5}\sin\theta_{5}\right)+L_{6}\left({{\dot{\theta }}_{6}}^{2}\cos\theta_{6}+{\ddot{\theta }}_{6}\sin\theta_{6}\right)-\frac{L_{7}}{2}\left({{\dot{\theta }}_{7}}^{2}\cos\theta_{7}+{\ddot{\theta }}_{7}\sin\theta_{7}\right)\right],\\ F_{7y}-F_{6y}-m_{7}g=m_{7}\left[L_{5}\left(-{{\dot{\theta }}_{5}}^{2}\sin\theta_{5}+{\ddot{\theta }}_{5}\cos\theta_{5}\right)+L_{6}\left(-{{\dot{\theta }}_{6}}^{2}\sin\theta_{6}+{\ddot{\theta }}_{6}\cos\theta_{6}\right)+\frac{L_{7}}{2}\left(-{{\dot{\theta }}_{7}}^{2}\sin\theta_{7}+{\ddot{\theta }}_{7}\cos\theta_{7}\right)\right],\\ M_{7}-M_{6}+F_{7x}\frac{L_{7}}{2}\sin\theta_{7}-F_{7y}\frac{L_{7}}{2}\cos\theta_{7}+F_{6x}\frac{L_{7}}{2}\sin\theta_{7}-F_{6y}\frac{L_{7}}{2}\cos\theta_{7}=I_{7}{\ddot{\theta }}_{7} \end{array}\right. \end{array}$$

in which all the variables are as defined in Figure 1.

### 2.4. Statistical Analysis

For all cats at each jump height, the magnitudes of the peak vertical ground reaction forces (*F*_*y*1_ and *F*_*y*2_), joint ranges of motion (ROM_1–6_), buffering durations (*t*_1_ and *t*_2_), joint moments (*M*_1–3_ and *M*_5–7_), joint reaction forces (*F*_1–3_ and *F*_5–7_), and joint work, as defined in Figure 1, were analyzed using an analysis of variance (ANOVA). An *F*-test was performed to determine the statistical significance of the test data at *p* of 0.05.

## 3. Results

### 3.1. Vertical Ground Reaction Forces

**Figure 3:**
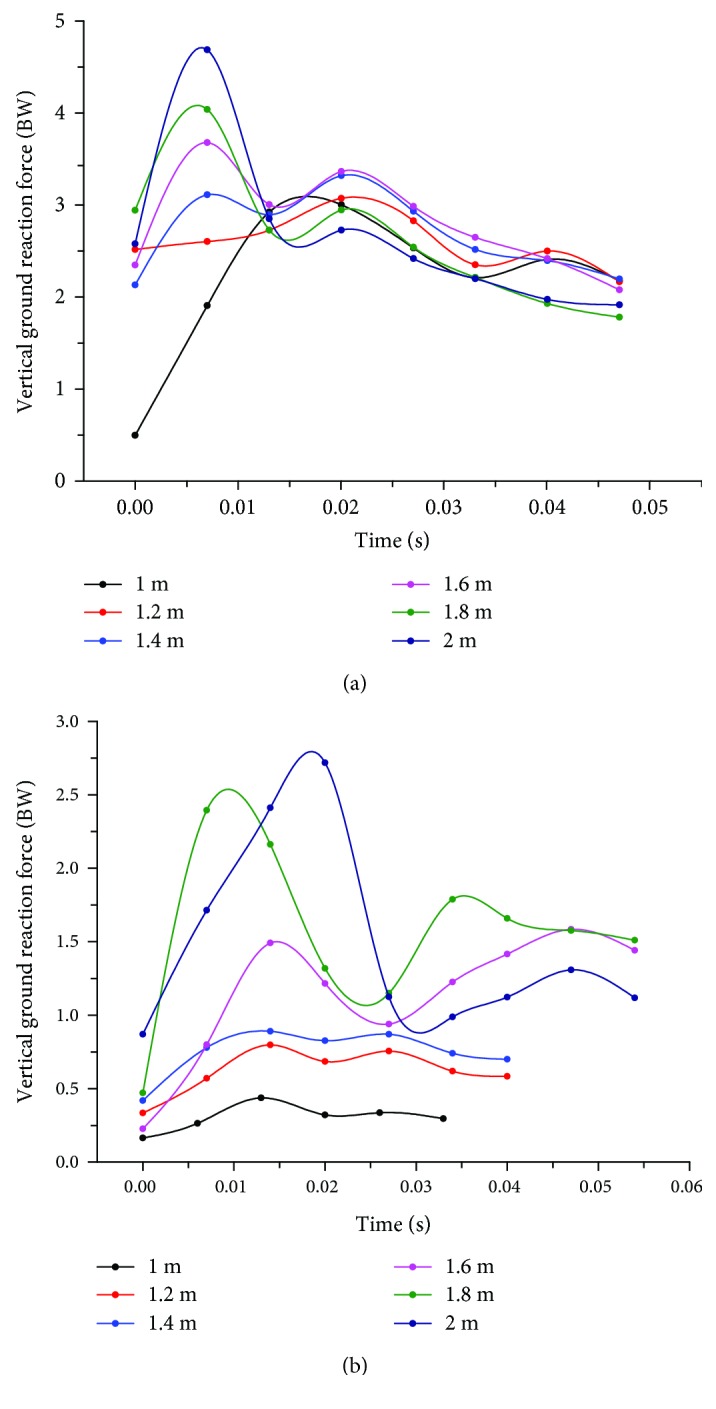
Representative average VGRF curves of the (a) RF and (b) RH limbs during the landing period from all jump heights.

A summary of the VGRFs of the RF and RH limbs is provided in Table 1 and graphically presented in Figure 3. Obviously, the peak VGRFs (*F*_*y*1_ and *F*_*y*2_) increased significantly (*p* < 0.05) with increasing jumping height. Double-peak patterns were also found at all jump heights, which was consistent with the findings of a previous study [15]. In a departure, however, the peak VGRFs of the RF limbs (*F*_*y*1_) were always significantly (*p* < 0.05) greater than those of the RH limbs (*F*_*y*2_) when the jump height was less than 2 m. However, the ratio of the peak VGRF of the RH limb to the total force increased with the increase in jump height, indicating that the hindlimbs experience a greater peak VGRF than the forelimbs when the cat jumps from a higher height.

### 3.2. Kinematics

The buffering durations of the RF and RH limbs and the time interval between the touchdown of the fore paws and that of the hind paws for different jump heights are shown in Table 2, while associated joint ROMs are shown in Table 3. In Table 3, ROM_1–6_ indicate the ranges of motion of the angle between the fore paw and the ground, wrist joint, and elbow joint and the angle between the hind paw and the ground, ankle joint, and knee joint, respectively. Because the angle between the fore paw and the ground eventually becomes zero during the landing process, ROM_1_ equals the initial angle at which the fore paw lands. Thus, as the jump height increases, the decreasing value of ROM_1_ indicates that before a cat jumps, it makes a subjective judgment to adjust the initial angle of its fore paw landing according to the jump height. Although no significant differences (*p* > 0.05) were found in ROM_2_ for any jump heights, other ROM values decreased significantly (*p* < 0.05) as the jump height increased.

We also analyzed the buffering durations, finding that there were no differences (*p* > 0.05) in *t*_1_ for all jump heights but that *t*_2_ increased significantly (*p* < 0.05) with increasing height. Additionally, the time interval between the touchdown of the fore paws and that of the hind paws also decreased with jump height.

**Figure 4:**
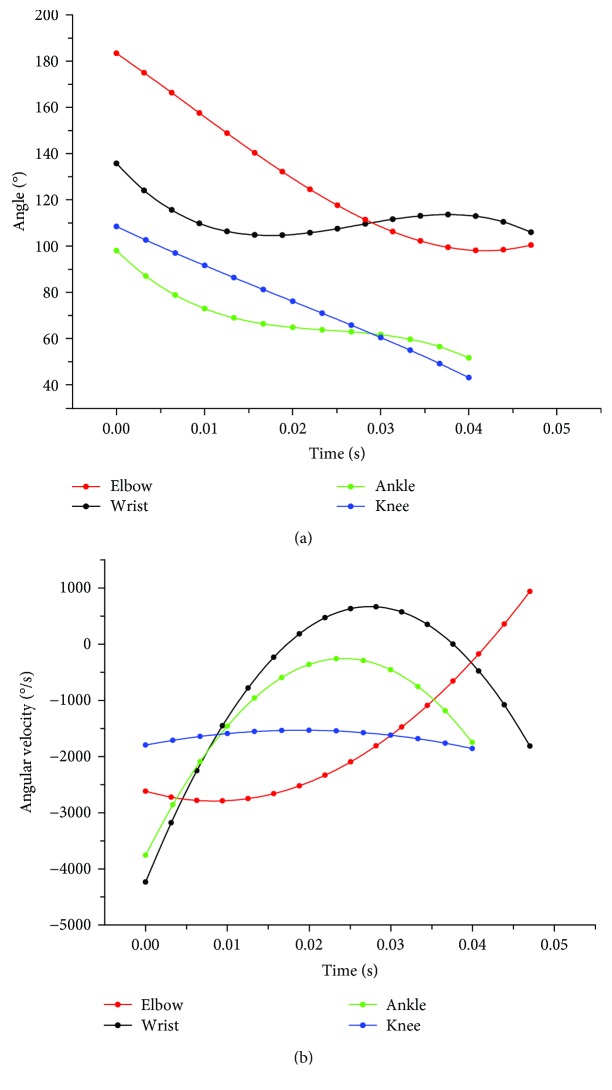
(a) Angles and (b) angular velocities of the wrist, elbow, ankle, and knee joints during landing from a 1.4 m jump.

In order to investigate the synergistic mechanism of cat joints, the values of angular velocity (deg/s) and angle (deg) for the wrist, elbow, ankle, and knee joints during a 1.4 m jump down were plotted as shown in Figure 4. Similar patterns of change were found across all cats for all jump heights. During the landing phase, the elbow, ankle, and knee joints underwent continuous flexion while the wrist joints experienced flexion, extension, and then flexion again. The maximum angular velocity of the wrist joint was reached at the beginning of the landing, as was also observed in the angular velocity curve of the ankle joint. As shown in Figure 4, the flexion velocity of the elbow joint manifested as a singular upward slope to its peak, while the angular velocity curve of the knee joint exhibited a generally downward opening with a peak in the middle of the buffering duration.

### 3.3. Kinetics

**Figure 5:**
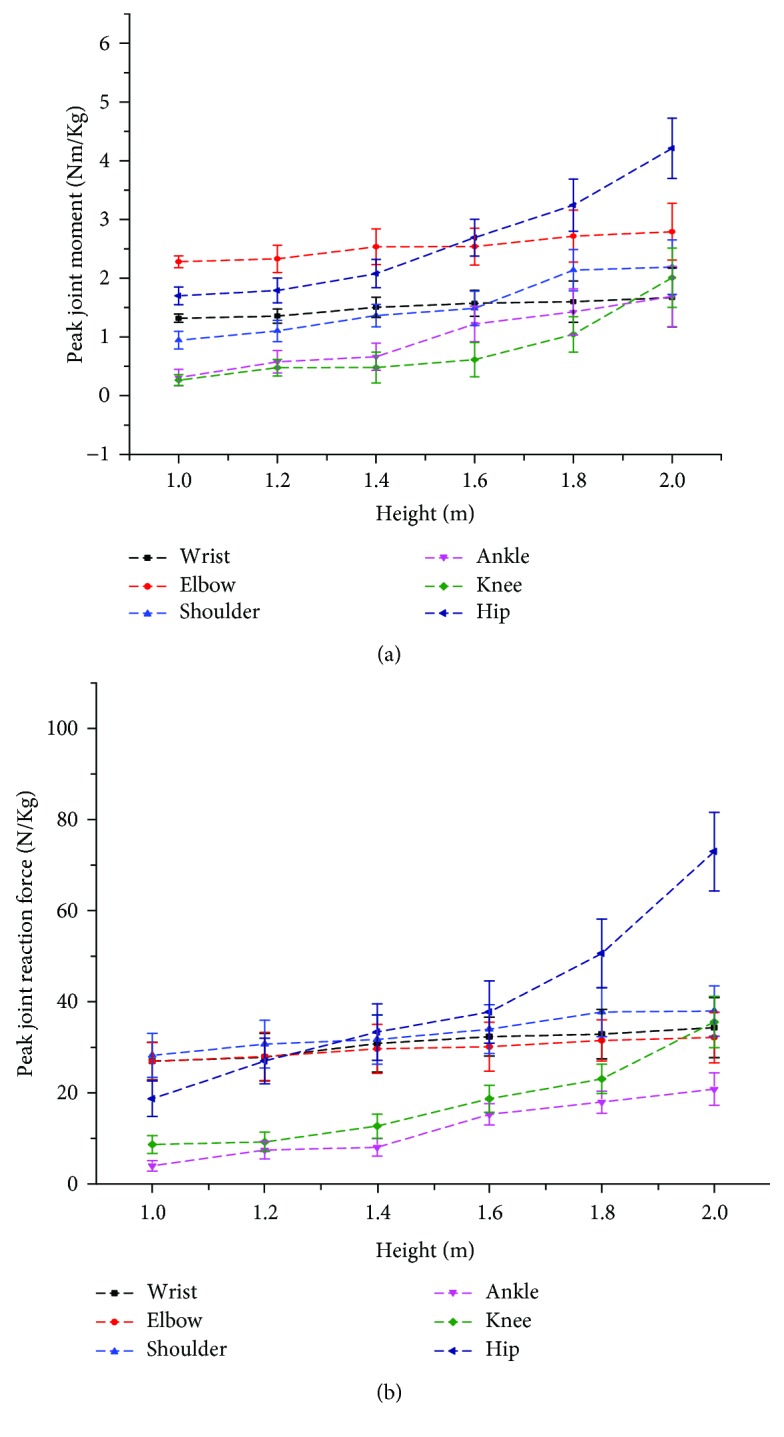
Mean and SD of the peak (a) joint moment and (b) joint reaction force versus jumping height for each joint during landing.

**Figure 6:**
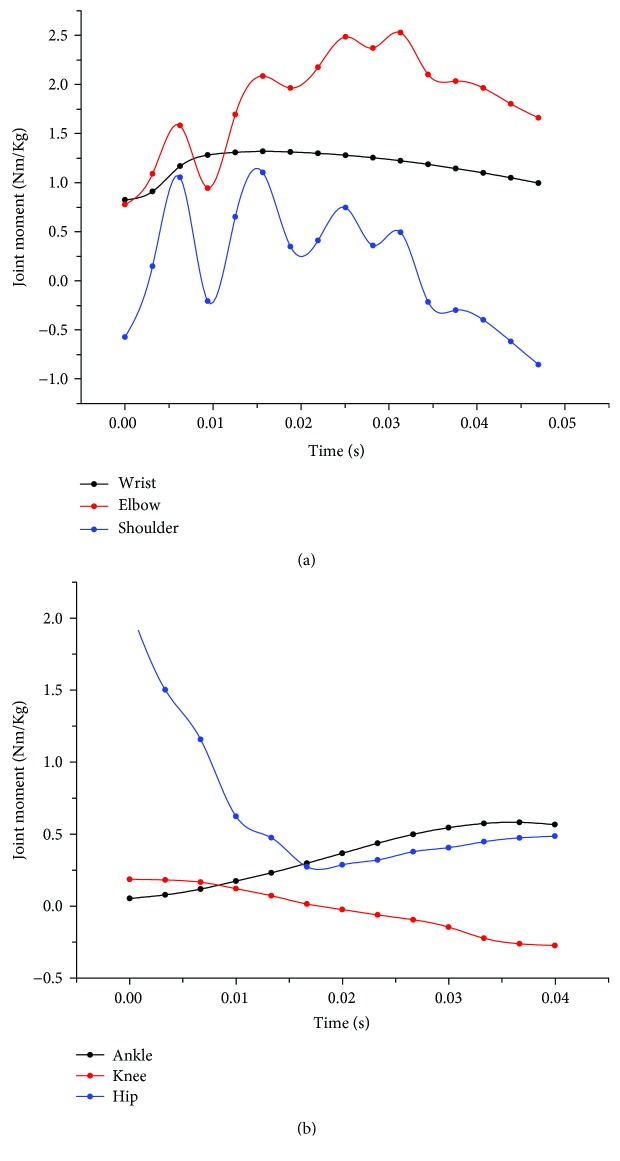
Joint moment in (a) the RF wrist, elbow, and shoulder joints and (b) the RH ankle, knee, and hip joints during landing from a 1.2 m jump.

The peak joint reaction forces, calculated as the resultant forces in the X and Y directions, and the joint moments are presented in Figures 5 and 6, respectively, in which it can be seen that the overall trend of the peak joint moment and the reaction force acting at each joint increases with increasing jump height. In the forelimbs, the peak elbow moment was significantly greater (*p* < 0.05) than that of the other two joints; however, there were no differences (*p* > 0.05) in the peak joint reaction forces at any of the three joints. Characteristically, although there were differences in the value and direction of the elbow and shoulder moments, their variation patterns were remarkably similar, showing multiple distinctive peaks. Only one peak was found in the wrist moments, where the variation was relatively small. In the hindlimbs, the peak moment of the hip joint was significantly greater (*p* < 0.05) than that of the ankle and knee joints, and the same was true for the joint reaction force. The joint moments of the ankle and knee increased but in opposite directions. A single significant peak and valley was observed in the hip joint moment curve during the impact phase of landing. Additionally, it can be observed that the joint moments and reaction forces acting on the joints of cats are of the same order of magnitude as that of humans, which is extremely large relative to the body size of cats, indicating that the synergistic mechanism employed by cat joints can indeed help to dissipate relatively tremendous impact forces.

**Figure 7:**
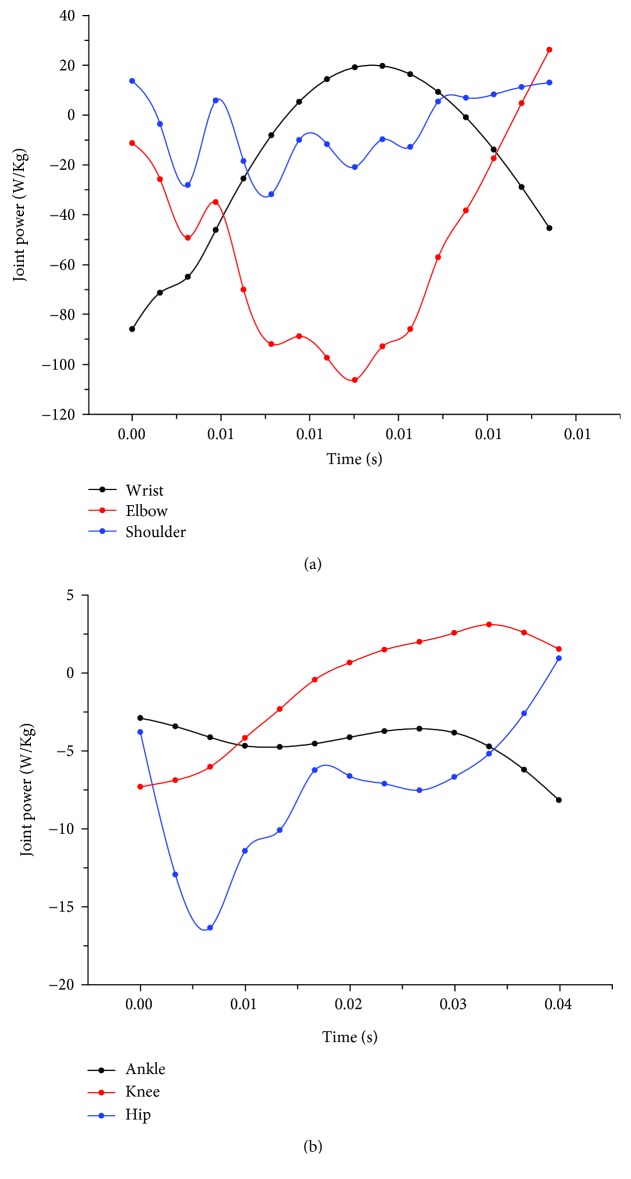
Joint power of (a) the RF wrist, elbow, and shoulder joints and (b) the RH ankle, knee, and hip joints during landing from a 1.2 m jump.

**Figure 8:**
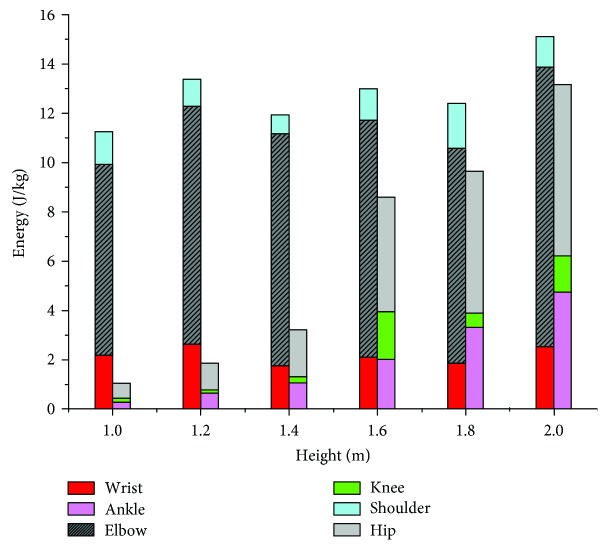
Mean joint work values for the wrist, elbow, shoulder, ankle, knee, and hip.

Joint power and work represent the maximum effort exerted by certain muscle groups during energy absorption. Similar variation patterns, showing multiple distinctive peaks, were present in both the elbow and shoulder joint power curves. A single peak was observed in the wrist power curve similar to the wrist moment curve. The values of hip power varied more than those of the knee joint and ankle joint (Figure 7). The means and standard deviations of joint work are provided in Table 4 and graphically represented in Figure 8. Although the forelimb was found to absorb more energy when the jumping height was less than 2 m, the ratio of energy absorbed by the hindlimb to the total energy increased with the increase in landing height. Therefore, it can be speculated that the hindlimb plays a greater role in the dissipation of energy as the jump height increases. Meanwhile, all cats utilized the elbow as the primary joint absorbing energy during the buffering durations of the forelimbs, and the hip joints in the hindlimbs provided greater relative contributions to overall energy absorption.

## 4. Discussion

### 4.1. Synergistic Mechanism of Cat Limb Joints

There have been several studies describing the body posture and muscle activity of cats in the take-off phase of a typical jump down. However, the synergistic mechanism of cat limb joints in the landing phase was still unclear. The results of this study suggest that there are general increases in the peak VGRF and the ratio of the peak VGRF in the RH limb to the total force with increasing jumping height. Although the VGRF in the RF limb was greater when the cats jumped from a height less than 2 m, it is logical to argue that the hindlimb will be utilized as the dominant limb to attenuate landing impulses from a jump of greater height, explaining the observed phenomenon that fractures in the hindlimb (61.5%) are more likely to occur than those in the forelimb (38.5%) in falling cats [1]. Moreover, double-peak patterns were manifested in the VGRFs of both the forelimb and hindlimb. Obviously, the first peak is due to the transmission of the accumulated downward momentum of the cat's mass after its paw touches the MatScan. We suspect that the second peak in the VGRFs of the forelimb is due to the rotation of the spine, suggesting that the forelimb actively shares the force in the hindlimb. We also infer that the second peak in the VGRFs of the hindlimb is due to the general forward movement after a cat has finished cushioning its landing.

In general, landing conditions are the best predictors of body dynamics throughout locomotion [16]. Accordingly, we further investigated the ROMs, buffering durations, and time intervals between the touchdown of the fore paws and that of the hind paws. Based on these results, we found that when jumping from different heights, a cat will make subjective judgments to adjust the speed of spinal rotation and the initial angle between the fore paw and the ground to ensure no significant difference in the buffering durations of the forelimbs. The way cats land (forelimbs land before hindlimbs) seems to be comparable to skipping gaits. In a recent study [17] on human skipping on uneven ground, the authors found that the trailing leg touched the ground with a flatter leg angle for a lowered touchdown surface, suggesting that the subjects were aware of the perturbation and lowered their center of mass in preparation for the drop. The comparison of results indicates that both cats and humans at least somewhat actively control and adapt the parameters of the leg that lands first after the flight phase. Notably, in human skipping gaits, the knee and ankle joint angles at the touchdown of the leading leg were not found to change between even and uneven conditions. However, in our results, the hindlimbs of cats were found to dissipate the greater impact force mainly through a larger joint ROM (attributable to increased buffering durations and joint ROMs with higher jumping height). It is logical to argue that adjustments of the trailing leg (forelimb) and coupled control with the leading leg (hindlimb) enhance stability and robustness, which may be the reason why these gaits are used in experimental situations by both humans and cats.

We also analyzed the angles and angular velocities of the wrist, elbow, ankle, and knee joints, finding that the angles of all joints except the wrist gradually decreased with increasing time after contact. As can be seen from the change in the wrist angle, the wrist joint serves more of a support and rotation function in the process of forelimb cushioning, spinal rotation, hindlimb cushioning, and forward movement, rather serving to absorb energy. The angular velocities of the joints were large at the initial stage of contact and then tended to decrease. Additionally, the angular velocity variations of different joints were not completely the same, but the same joint did show similar changes under different landing heights. These results suggest that a generalized motor program can be adopted to activate limb joints for different landing heights. In the future development of related exoskeletal equipment, these kinematic data can be combined to drive the equipment and achieve a cat-like buffering mechanism that allows limb joints to reduce mechanical energy and improve energy absorption efficiency.

### 4.2. Dominant Limb Joint Muscle Groups in Energy Absorption

Energy flows give rise to a variety of forms of movement that would not have taken place without them. The only source of energy generation and the major site of energy absorption in all living things are the muscles, as only a small portion of energy is dissipated by joint friction and connective tissue adhesion. Therefore, it can be considered that energy continuously flows into and out of the limb muscles between each limb segment. Here, using inverse dynamics, we calculated the joint moments, joint reaction forces, and joint powers, then quantified the contributions of the limb joints to energy absorption when landing from different jump heights.

As can be seen from the joint moment and joint power curves of Figures 6 and 7, in the early phase of forelimb landing, there is a process of energy transfer from the wrist to the elbow and shoulder. However, in Figure 7, it can be seen that the power curve of the shoulder fluctuates up and down around a joint power value of 0, indicating that most of the energy is absorbed by the elbow. We believe that the shoulder serves more of a weight-bearing and rotation function during landing similar to that of the wrist. Differently, the hip absorbs more energy in the early phase of hindlimb landing because some of the energy is transferred to the hip by the rotation of the spine. In the late phase of hindlimb landing, the ankle starts to produce some energy, which we theorize because the cat is beginning to get up and move forward.

The magnitudes of the peak joint moment and peak joint reaction force experienced by each cat in response to increasing jump height indicate that cats tend to distribute the greater demands from higher jump heights to the elbow and hip. Compared to the maximum joint moments in a study of human landing [18], the moments in the cats' joints are relatively large. One reason cats do not experience injury as readily is that cats can control joint motion and attenuate the impact force experienced during landing in accordance with the synergistic mechanism described in the previous section. Another reason we suspect for this resistance to injury is that the microstructures of cat bones, especially their claws, are beneficial for avoiding impact injuries. However, further studies on cat anatomy and micro-CT scanning are still required. It is worth noting that because we simplified the jump down of each cat as a two-dimensional motion in the sagittal plane, the forces in the mediolateral direction were neglected. Additionally, the peak forces in the fore-aft direction were only 2–4% of the peak VGRFs (from unpublished data of this study) and their directions should be backward to steady the paws as they strike the ground. Based on equations (2)–(7), this assumption leads directly to an increase in *F*_1*x*_ and *F*_2*x*_, which will always be negative, resulting in a decrease in the negative values of *M*_1_ and *M*_2_. However, the exact effects of this assumption on other joints cannot be determined because the signs of the joint reaction forces and joint moments are not constant.

Biarticular muscles generate moments at both joints the muscles cross and are used to transport mechanical energy during locomotion [19–21]. A number of studies have been conducted on the muscle activities in the elbows of cats during various forms of locomotion [10, 22, 23], and it has been suggested that the long head of the triceps and biceps, both biarticular muscles, play a major role during landing. The biceps is a fusiform muscle in the front of the humerus and the long head of the triceps, triangular in shape, connects the scapula to the olecranon. Importantly, both have long tendons and the muscle fascicules are arranged in a penniform shape, which is well suited for the dissipation of energy [24, 25]. In the hindlimbs, the feline hamstring muscle group (biceps femoris, semitendinosus, and semimembranosus) has a larger mechanical advantage at the hip [20]. The biceps femoris is a large flat muscle that covers two-thirds of the lateral side of the femur, the semitendinosus is a slender muscle with thin and firm tendons, and the semimembranosus has the same shape as the long head of the triceps, with firm and flat tendons. This arrangement allows energy to be transported from segment to segment and then be absorbed by the hip extensors.

## 5. Conclusions

Using the principle of inverse dynamics and summarizing the results of cat landing experiments, we are able to explain how cats control joint motion to dissipate impact force and to analyze the joint energy absorption strategies employed during landing, gaining insight into the internal buffering mechanism. Our results show that cats can adopt a general mechanism of limb movement that is quite beneficial in attenuating impact force. Notably, the elbow and hip muscle groups were found to be dominant in energy absorption. The results of this study can provide biological inspiration for high-efficiency buffering and energy-absorption equipment to reduce landing fall injuries in humans. It should be noted that, in this study, we simplified the jump down of each cat as a motion in a two-dimensional plane, as in previous studies, while in fact, during the jump, the movement planes should be divided into the sagittal, coronal, and transverse planes, so further study is warranted to capture the effects of movements in these directions.

## Figures and Tables

**Table 1:** Mean and SD of peak VGRFs and ratio of *F*_*y*2_ to total force (*F*_*y*1_ and *F*_*y*2_) for each jump height^a^.

Jump height (m)	*F*_*y*1_ (N/kg)^∗^	*F*_*y*2_ (N/kg)^∗^	Ratio (%)
1.0	29.44 (8.39)^b^	4.27 (1.64)	12.67
1.2	30.13 (9.17)^b^	7.77 (2.16)	20.49
1.4	32.53 (9.36)^b^	8.72 (2.58)	21.14
1.6	36.05 (10.21)^b^	13.04 (4.12)	26.57
1.8	39.58 (11.05)^b^	20.43 (5.22)	34.04
2.0	45.94 (11.34)^b^	26.64 (7.41)	36.70

^a^Values in the parentheses are the standard deviations (SD). ^b^Significantly different from *F*_*y*2_ at the same height. ^∗^Parameter shows statistically significant difference between jump heights.

**Table 2:** Means and SD of the buffering durations of the RF (*t*_1_) and RH (*t*_2_) limbs, in a time interval (Δ*t*) between the touchdown of the fore paws and the hind paws, for different jump heights^a^.

Jump height (m)	*t*_1_ (ms)	*t*_2_ (ms)^∗^	Δ*t* (ms)
1.0	47.29 (1.56)	33.67 (4.27)	60.00 (8.22)
1.2	46.18 (2.28)	40.33 (3.62)	30.26 (6.31)
1.4	48.67 (1.09)	42.28 (1.91)	27.57 (5.24)
1.6	47.33 (3.62)	53.67 (2.13)	23.33 (7.10)
1.8	46.96 (1.33)	57.00 (5.84)	17.42 (8.99)
2.0	48.21 (2.77)	59.19 (4.38)	−10.00 (10.57)

^a^Values in the parentheses are the standard deviations (SD). ^∗^Parameter shows statistically significant difference between jump heights.

**Table 3:** Mean and SD of the selected ROMs across jump heights^a^.

Height (m)	ROM_1_ (deg)^∗^	ROM_2_ (deg)	ROM_3_ (deg)^∗^	ROM_4_ (deg)^∗^	ROM_5_ (deg)^∗^	ROM_6_ (deg)^∗^
1.0	52.03 (7.28)	49.86 (8.96)	68.25 (11.93)	15.84 (3.91)	30.64 (10.36)	37.13 (8.09)
1.2	47.83 (8.35)	34.16 (6.31)	78.49 (12.54)	20.51 (5.15)	41.22 (8.69)	56.75 (12.67)
1.4	45.82 (6.27)	31.35 (6.53)	80.93 (13.10)	26.51 (6.44)	42.52 (9.42)	61.70 (14.95)
1.6	39.99 (7.99)	30.41 (5.08)	88.90 (14.97)	32.61 (5.98)	43.40 (11.67)	73.90 (15.38)
1.8	37.18 (5.84)	32.97 (9.76)	90.12 (15.75)	34.29 (8.16)	43.62 (10.20)	74.07 (19.17)
2.0	36.08 (4.18)	46.08 (6.30)	97.76 (15.35)	46.70 (9.76)	70.89 (17.09)	120.26 (23.63)

^a^Values in the parentheses are the standard deviation (SD). ROM_1–6_ are the ranges of motion of the angle between the fore paw and the ground, wrist joint, and elbow joint and the angle between the hind paw and the ground, ankle joint, and knee joint. ^∗^Parameter shows statistically significant difference between jump heights.

**Table 4:** Mean and SD of energy absorbed by the RF and RH limb joints across all jump heights^a^.

Height (m)	Wrist (J/kg)	Elbow (J/kg)^b^	Shoulder (J/kg)	Ankle (J/kg)	Knee (J/kg)^c^	Hip (J/kg)
1.0	2.1696 (0.65)	7.7547 (1.19)	1.3223 (0.34)	0.2714 (0.04)	0.1552 (0.02)	0.61 (0.06)
1.2	2.6243 (0.78)	9.661 (2.01)	1.0907 (0.28)	0.6408 (0.09)	0.1184 (0.01)	1.0944 (0.30)
1.4	1.7404 (0.49)	9.4252 (1.89)	0.7686 (0.16)	1.0496 (0.22)	0.2508 (0.04)	1.9049 (0.61)
1.6	2.1053 (0.51)	9.6083 (2.34)	1.2798 (0.33)	1.9996 (0.56)	1.9437 (0.40)	4.6483 (0.90)
1.8	1.8471 (0.37)	8.7272 (1.88)	1.8247 (0.42)	3.3068 (0.90)	0.5844 (0.07)	5.7561 (1.08)
2.0	2.524 (0.44)	11.352 (2.70)	1.2378 (0.35)	4.7424 (1.11)	1.4591 (0.41)	6.9656 (1.23)

^a^Values in the parentheses are the standard deviation (SD). ^b^Parameter shows a statistically significant difference from the wrist and shoulder at each height. ^c^Parameter shows a statistically significant difference from the ankle and hip at each height.

## Data Availability

All data included in this study are available upon request by contact with the corresponding author.
